# Analysis of purple urine bag syndrome by low vacuum scanning electron microscopy

**DOI:** 10.1007/s00795-022-00313-0

**Published:** 2022-02-04

**Authors:** Makoto Abe, Masahito Furuichi, Toshihiko Ishimitsu, Akihiro Tojo

**Affiliations:** Department of Nephrology and Hypertension, Dokkyo Medical University, 880 Kitakobayashi, Mibu, Tochigi 321-0293 Japan

**Keywords:** Purple urine bag, Urinary sediment, Bladder catheter, Low vacuum scanning electron microscope

## Abstract

Purple urine bag syndrome (PUBS) is seen in the prolonged indwelling bladder catheters, and the mechanism of its onset was investigated using low vacuum scanning electron microscopy (LVSEM), which enables us to study the 3D structure of urinary sediments and urine bag walls. The urinary sediment and urine bags of 2 cases of PUBS were observed by LVSEM. The urine was brown turbid urine with a pH of 8.5, and magnesium phosphate stones and granules were observed in the urinary sediment together with Gram-positive and Gram-negative bacilli. Bacteria that moved by Brownian motion were observed with a dark-field microscope. LVSEM showed granular crystals around the bacilli, cocci, or mycelium that adhered to the walls of the bag. Granular crystals were dissolved in chloroform and presumed to be a mixture of the bacterial metabolites indigo blue and indirubin red. LVSEM also detected unusual tubular and honeycomb-like graphene in the urinary sediments, which were derived from the inner layer of the silicon elastomer-coated rubber catheter. LVSEM revealed purple crystals produced by bacteria or fungi attached to the urine bag that caused PUBS.

## Introduction

Purple urine bag syndrome (PUBS) is rarely observed in the patients with long-term balloon catheter placement [1, 2]. Some bacteria, such as *Klebsiella*, are thought to breakdown indoxyl sulfate to produce blue indigo and red indirubin pigments [3]. However, it is not completely understood why it is only found in some patients and why the urine itself does not turn purple [4]. Therefore, we tried to elucidate the mechanism of PUBS using low vacuum scanning electron microscopy (LVSEM).

LVSEM is a nonperturbing technology that requires minimal sample preparation [5, 6]. LVSEM using paraffin-embedded slide glass specimens with periodic acid Schiff methenamine silver staining is useful for observing renal biopsy samples to identify electron-dense deposits and changes in the glomerular basement membrane and podocytes [5–8]. Even though the resolution of LVSEM is not sufficient at more than × 10,000 magnification, black and white reversed images of LVSEM images become similar to transmission electron microscopy (TEM) images in the observation of renal biopsy samples [6]. Thus, LVSEM could be a substitute for TEM, for which special preparation and skill, as well as time-consuming processes, are required to obtain images. Moreover, LVSEM has facilitated a new understanding of various types of 3D structures including those observed in regenerated organs, vascular injury thrombus formation, and tumor cells [9].

In the present study, we applied LVSEM in the observation of urinary sediments, urine bags, and bladder catheters to understand the mechanism of purple urine bag syndrome.

## Methods

Urine samples (10 mL) from patients with purple urine bag syndrome were centrifuged at 500×*g* for 5 min. After removing the supernatant by decantation, 200 μL of urinary sediments were stained with 20 μL of Sternheimer stain solution (Muto Pure Chemicals Co., Ltd., Tokyo, Japan) and observed with a dark-field microscope. Some urinary sediments were stained with a Gram staining kit (Fujifilm, Osaka, Japan). The remaining urinary sediments stained with Sternheimer stain solution were washed with 1000 μL of distilled water twice. Fifteen microliters of urinary sediments was mounted on a carbon filter membrane (Nisshin EM, Tokyo, Japan) and observed by LVSEM (Hitachi TM4000 Plus, Tokyo, Japan). The acceleration voltage was fixed at 15 kV with a vacuum of 30 Pa to detect backscattered electrons [6]. Urine samples were collected from urine bags with the approval of the research ethics committee of Dokkyo Medical University (R-2-1).

The walls of the urine bag from cases of purple urine bag syndrome were cut and directly observed by LVSEM. To analyze the properties of the crystals attached to the urine bag, the wall surface of the purple bag was immersed in 1 N hydrochloric acid, 1 N sodium hydroxide, 30% hydrogen peroxide, and 99% chloroform, and the decolorization of the dye and the dissolution of the crystals were observed by LVSEM.

To confirm crystal formation from indoxyl sulfate by the bacteria on the urine bag surface, bacteria on the urine bag surface were cultured by sheep blood agar palate (Nippon Becton Dickinson Company Ltd, Fukushima, Japan) with 250 μmole/L indoxyl sulfate (Cayman Chemical Company, Ann Arber, MI, USA) in a CO_2_ incubator for 3 days. The surface of agar was transferred to the glass slides by stamping and observed by light microscopy.

A silicone elastomer-coated bladder catheter (Medicon Inc., Osaka, Japan) was cut, and both the inner and outer surfaces of the tube were stained with 1% Ponceau S solution (Olympus Optical Co., Ltd., Tokyo, Japan) for 15 min, washed with distilled water, mounted on a carbon filter, and observed by LVSEM.

## Results

**Fig. 1:**
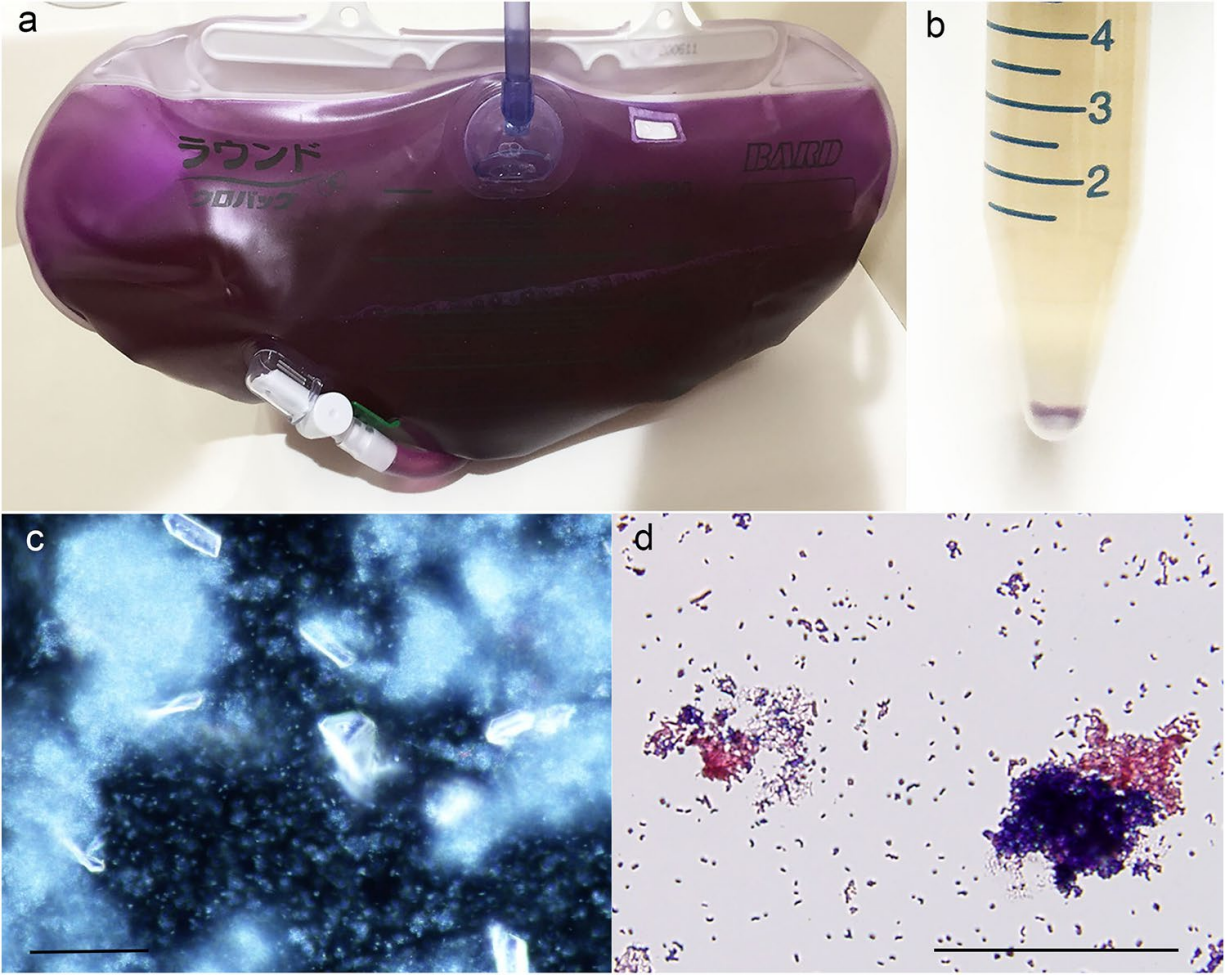
Case 1 of purple urine bag syndrome (**a**), urine after centrifugation (**b**), urinary sediments observed with dark-field microscopy (**c**), and Gram staining of urinary sediments (**d**). Even though the urine bag is purple (**a**), and urine itself is brown with purple crystals in the sediments (**b**) and with more than two species of bacteria (**c**, **d**). The bars indicate 50 μm

Table 1 shows the clinical background of the two cases of PUBS. In both cases, approximately 1 month after wearing the bladder balloon catheter, urinary tract infections were confirmed by an increase in urinary leukocytes, multiple bacteria, or fungi, but no antibiotics were given. In case 1, the urine bag was purple, but the color of the urine was brown, and when centrifuged, a purple substance is precipitated in the sediment (Fig. 1a, b). The supernatant was discarded, a part of the precipitate was stained with Sternheimer staining, and observed with a dark-field microscope to identify struvite crystals, which are magnesium ammonium phosphate stones, and white glowing coccid and bacilli showed Brownian motion (Fig. 1c). Gram staining of a part of the sediment revealed a mixture of red Gram-negative bacilli and blue Gram-positive cocci (Fig. 1d). In urine culture, the Gram-negative rods were *Enterobacter cloacae* and *Pseudomonas aeruginosa*, and the Gram-positive cocci were *Streptococcus anginosus* (Table 1).

**Table 1:** Clinical data of two cases of purple urine bag syndrome

	Case 1 85-yo male	Case 2 77-yo male
Background disease	Post-renal renal failure with BPH	Myocardial infarction
Urinary pH	8.5	5.5
Urinary sediments	RBC 5–9/HPF WBC 5–9/HPF Bacteria +	RBC 10–19/HPF WBC 30–49/HPF Bacteria +
Urinary culture	*E. cloacae* *P. aeruginosa* *S. anginosus*	*S. epidermidis* *C. albicans*
Blood WBC, CRP	7300/μL, 5.14 mg/dL	9900/μL, 1.82 mg/dL
Days after inserting the urine bag	28 days	32 days
Use of antibiotics	No	No

**Fig. 2:**
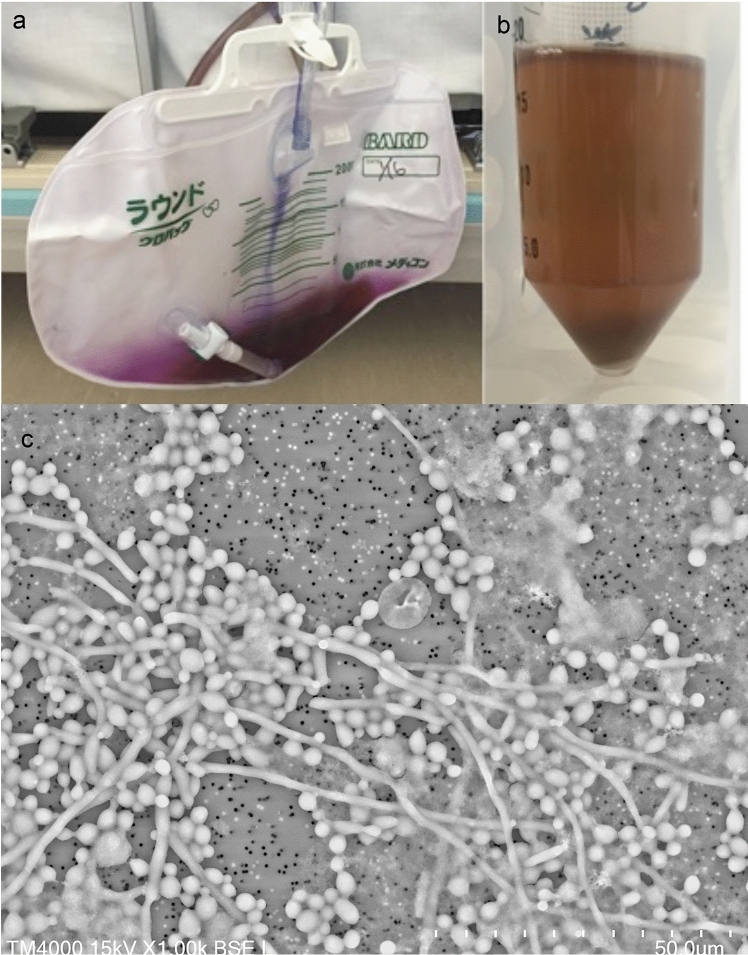
Case 2 of purple urine bag syndrome (**a**), urine after centrifugation (**b**), and urinary sediments observed by LVSEM (**c**). Urine in the purple bag (**a**) is not purple (**b**), and urinary sediments include fungi and bacteria (**c**). Magnification × 1000 (**c**). The bars indicate 50 μm

In case 2, the wall of the urine bag was purple, but the urine was brown (Fig. 2a, b). When the urine sediment was observed by LVSEM, fungal hyphae and large spherical yeast-shaped spores were observed, and smaller cocci were observed in the background (Fig. 2c). Urine culture revealed that the fungus was *Candida albicans* and the background cocci were *Staphylococcus epidermidis* (Table 1).

**Fig. 3:**
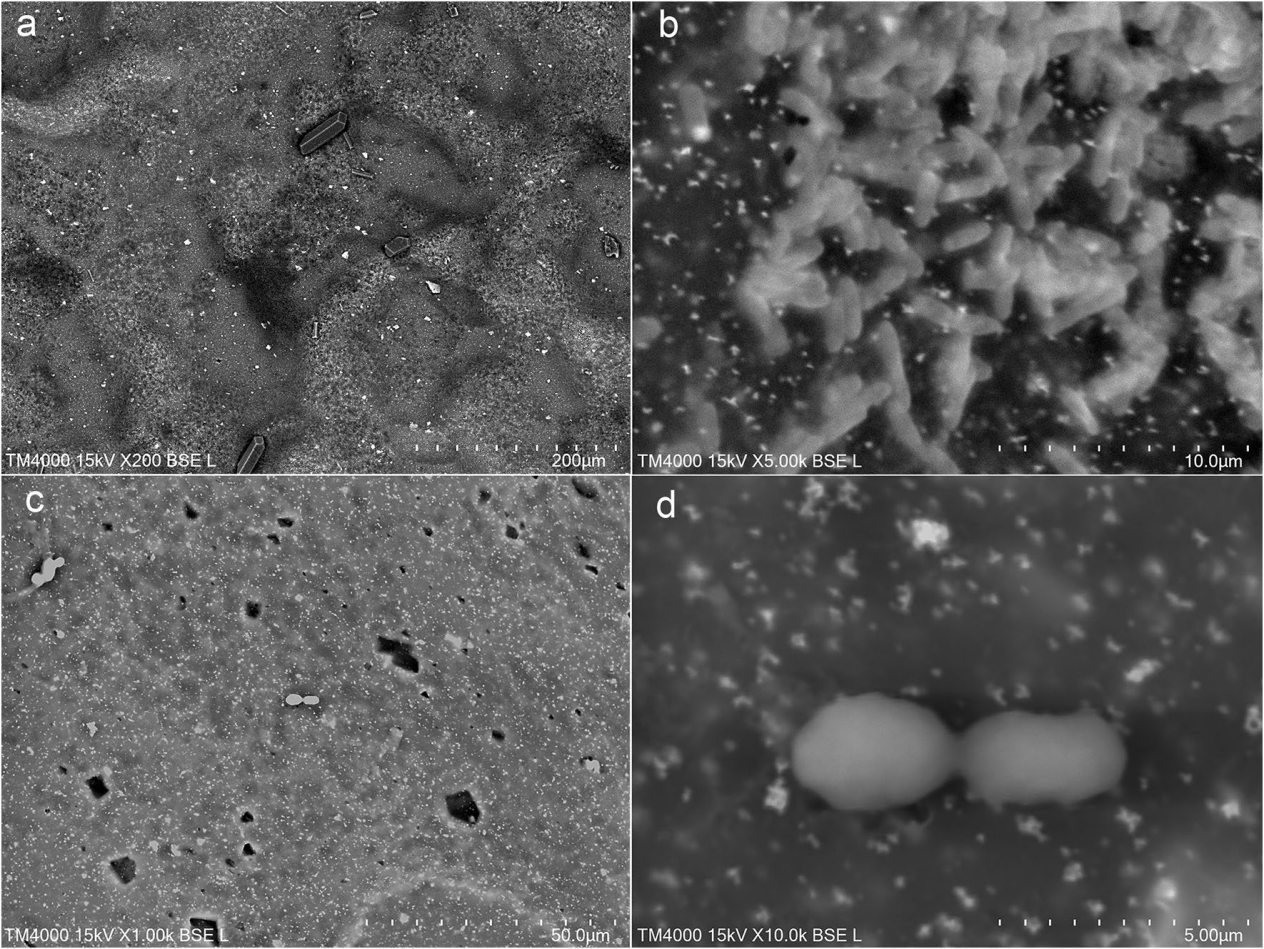
LVSEM observation of the inner surface of the purple urine bags in case 1 (**a**, **b**) and case 2 (**c**, **d**). Magnification × 200 (**a**), × 5000 (**b**), × 1000 (**c**), and × 10,000 (**d**) The bars indicate 200 μm (**a**), 10 μm (**b**), 50 μm (**c**), and 5 μm (**d**).

**Fig. 4:**
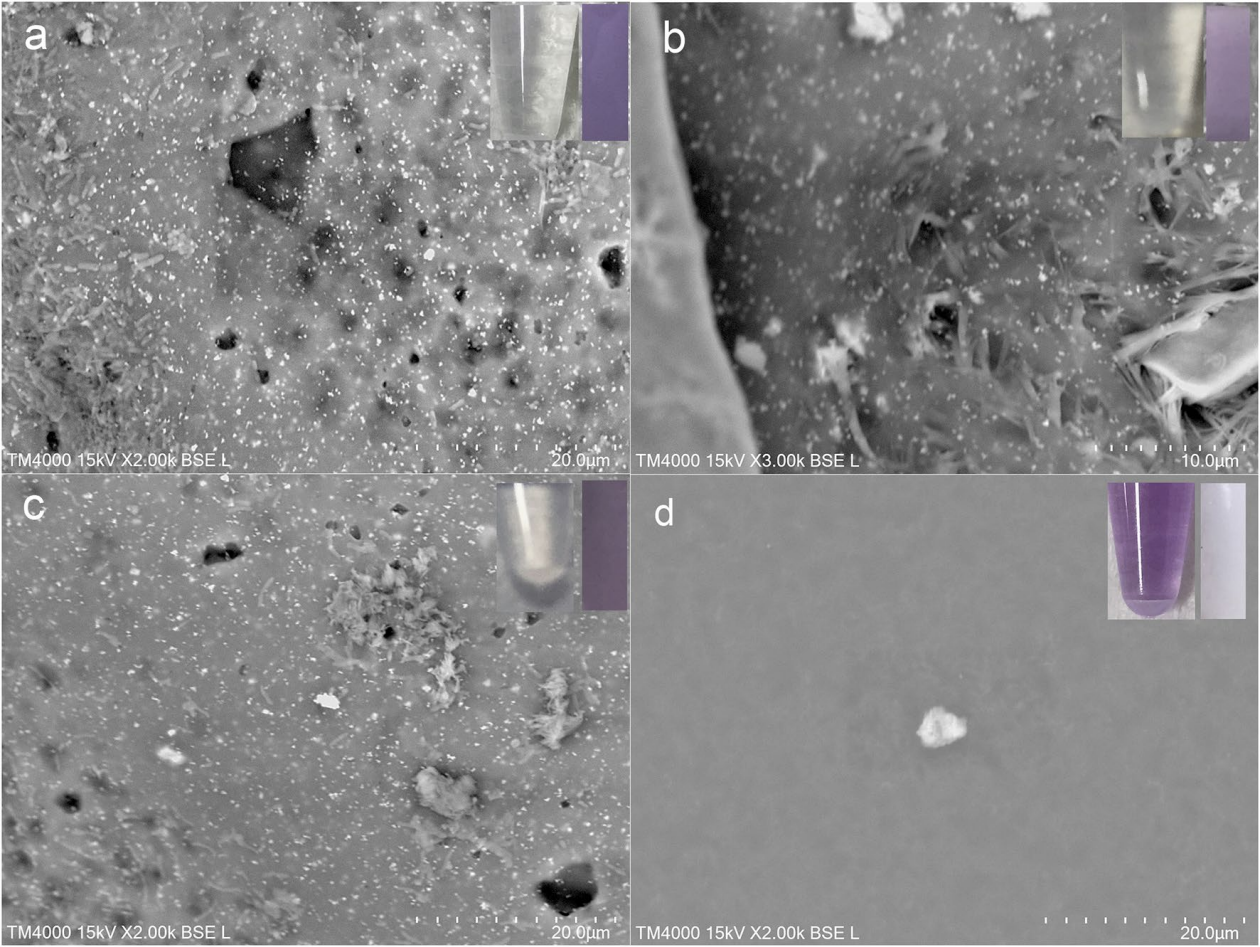
LVSEM observation of the segments of the surface of purple urinary bag in case 1 immersed in 1 N HCl (**a**), 1 N NaOH (**b**), 30% H_2_O_2_ (**c**), and chloroform (**d**). The insertions are the color of the immersion solution (left) and segment of purple urine bag (right) after 15 min. Magnification × 2000 (**a**, **c**, **d**) and × 3000 (**b**). The bars indicate 20 μm (**a**, **c**, **d**) and 10 μm (**b**)

**Fig. 5:**
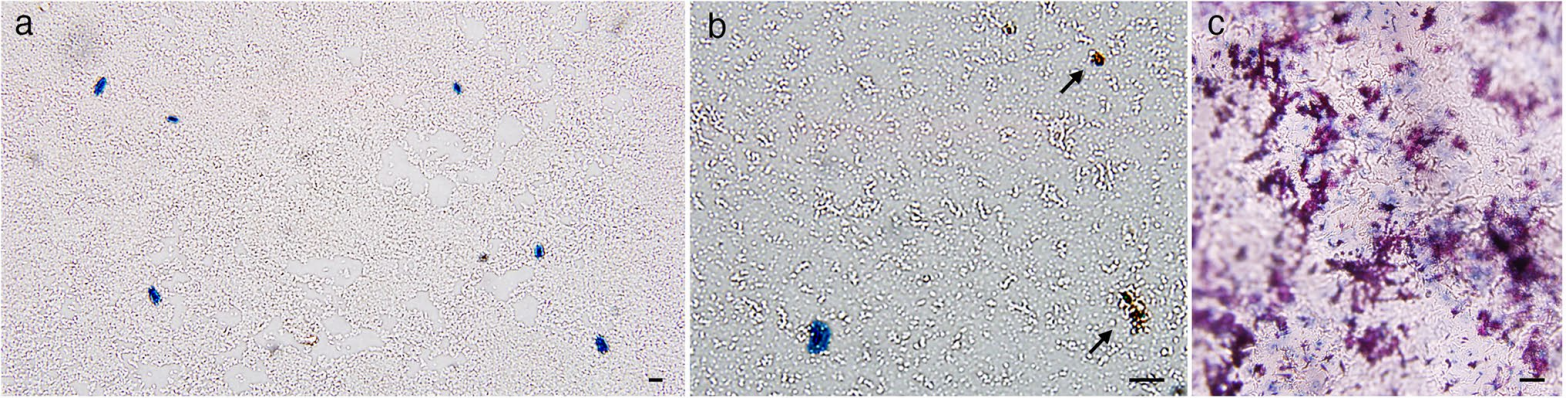
Light microscopic observation of crystals with blue and brown (arrow) colors formed from indoxyl sulfate via incubated bacteria from the urine bag of case 1 (**a**, **b**) and light microscopy of crystals on the lucid polypropylene surface of urine bag (**c**). The bacteria obtained from urine bags were incubated with indoxyl sulfate, and formed blue crystals (**a**) and brown crystals (**b**), which resembled the purple and blue crystals on the surface of urine bags (**c**). The bars indicate 10 μm (**a**–**c**)

LVSEM observations showed many amorphous crystals on the purple surface of the urine bag. In Case 1, bacilli adhered to the white polyvinyl chloride (PVC) surface of the urine bag, and many amorphous crystals were observed around them (Fig. 3a, b). In Case 2, LVSEM revealed yeast-like spores with large *C. albicans* adhered to the urine bag, and many irregularly shaped crystals were observed around them (Fig. 3c, d). The purple surface of the urine bag was cut into strips and soaked in 1 N HCL acid solution (Fig. 4a), 1 N NaOH alkali solution (Fig. 4b), 30% hydrogen peroxide (Fig. 4c), or chloroform (Fig. 4d) for 15 min to examine the bleaching of the urine bag and the dissolution of crystals. Chloroform easily decolorized the purple color of the urine bag, dissolved the crystals on the surface, and turned the extract into purple (Fig. 4d). On the other hand, the acidic solution did not elute the purple urine bag at all and granular crystals and bacteria remained on the wall (Fig. 4a). The alkaline solution (Fig. 4b) and the hydrogen peroxide solution (Fig. 4c) slightly dissolved with few bacteria but granular and spindle crystals remained on the wall in 15 min, and finally decolorized after a long time. To confirm that the bacteria on the bag produced colored crystals, bacteria on the urine bag were swabbed and incubated on a blood agar plate with indoxyl sulfate. Crystals with blue (Fig. 5a) and brown color (Fig. 5b) were identified around the bacteria. The crystals produced from indoxyl sulfate by cultured bacteria were not perfectly the same but similar to purple and blue crystals on the lucid polypropylene surface of the urine bag (Fig. 5c).

**Fig. 6:**
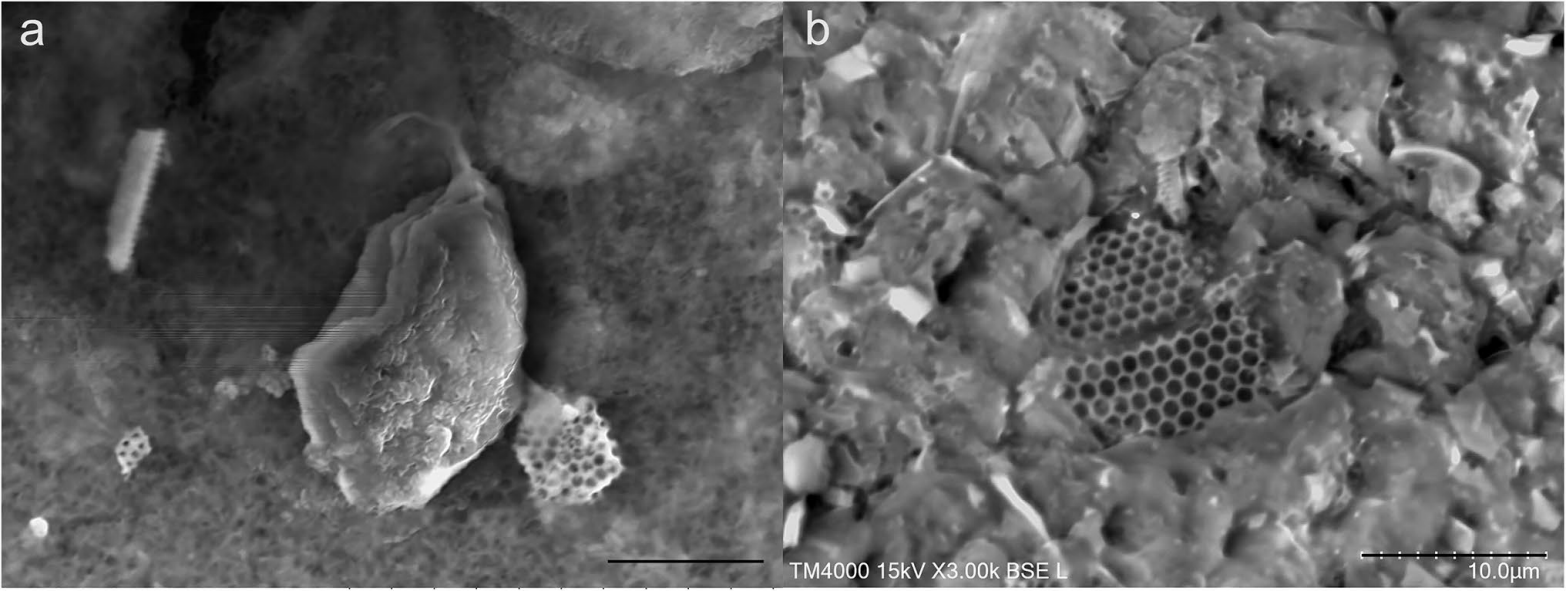
Urinary sediments (**a**) and the inner surface of the urinary bladder catheter (**b**) observed by LVSEM. Graphene and tubular structures (**a**) in the urinary sediments are derived from the surface of the urinary bladder catheter (**b**). Magnification × 3000 (**a**, **b**). The bars indicate 10 μm (**a**, **b**)

LVSEM observations of urine in the bag showed strange honeycomb and tubular structures with spikes, similar to graphene and carbon nanotubes (Fig. 6a). LVSEM observations of the bladder catheter revealed that these structures shed from the luminal wall of the bladder catheter coated with a silicon elastomer to reinforce the rubber tube (Fig. 6b).

## Discussion

In this case study, LVSEM observations showed that the urine bag was colored purple by the irregularly shaped crystals produced by the bacteria on the urine bag, even though the urine itself was brown in PUBS.

### Relationship between the characteristics of urinary bacteria and purple urine bags

Since purple urine bag syndrome was first reported in 1978 [1], it has been recognized as a complication of urinary tract infections (UTIs) wherein urine bags turn purple, and it occurs predominantly in female, chronically catheterized patients with alkaline urine [2] because of frequent UTIs in these cases. Some bacteria, including *Providencia stuartii* and *Providencia rettgeri*, *Klebsiella pneumoniae*, *Proteus mirabilis*, *Escherichia coli*, *Enterococcus species*, and *Morganella morganii*, produce sulfatase and phosphatase, which oxidize the uremic toxin indoxyl sulfate to indigo (a blue pigment) and indirubin (a red pigment), and these pigment mixtures react with the urine catheter and bag to produce a striking purple hue [3, 10, 11].

In case 1, *E. cloacae* and *P. aeruginosa* are Gram-negative bacilli with flagella and motility, and cannot produce indole. Indole is produced from food-derived tryptophan via a deamination reaction by intestinal bacteria, and is sulfated in the liver to indoxyl sulfate (indican), which is filtered by glomeruli and removed in urine. *Enterobacter* has an indoxyl phosphatase and produces indigo from indican [3], and *P. aeruginosa* has alkylsulfatase (SdsA1) [12], which decomposes indoxyl sulfate into indoxyl and further oxidizes it to indigo (blue) and indirubin (red) [10, 11]. *S. anginosus* is a Gram-positive coccus that has phosphoserine phosphatase, produces H_2_S, and may be involved in crystal formation [13]. Actually, bacteria on the bag in case 1 can produce blue- and brown-colored crystals from indoxyl sulfate. In Case 2, *S. epidermidis* and *C. albicans* were identified in urine cultures. *S. epidermidis* has phosphatase [14], and *C. albicans* has sulfatase [15], so indigo and indirubin can be produced from indoxyl sulfate.

### Identification of crystals by morphology and solubility

Purple amorphous crystals were found on the surface of the urine bag. Calcium oxalate, uric acid, cystine crystals, and magnesium ammonium phosphate crystals commonly found in urine are denied by morphology, but are also soluble in hydrochloric acid [16, 17]. Atypical phosphates and yellow urates are shown in the form of amorphous granules, but they are rejected, because they are dissolved in hydrochloric acid or acetic acid. Purple crystals are indigotin crystals that are difficult to dissolve in acids and alkalis, but they are soluble in chloroform [18]. This is consistent with the result in this case. Observations by LVSEM clearly showed that the purple color of PUBS was not the color of the liquid phase urine itself, but the color of the crystals that grew around the bacteria attached to the wall of the bag. Purple crystals are also seen in the precipitate of urine after centrifugation (Fig. 1b). The purple color of the urine bag disappeared after treatment with antibiotics [19].

### Identification of new structures in urinary sediment by LVSEM

Our study clearly demonstrated novel 3D structures in the urinary crystals by LVSEM. In contrast to conventional SEM [20–22], LVSEM does not require a freeze-drying process; thus, smaller crystals and bacteria are not lost during processing. The strange honeycomb-like structure and tubular structure with windows or spikes were similar to the graphene and carbon nanotubes in the silicon-polymer coating [23]. Stretchable graphene is used for various purposes on rubber structures, including light-emitting diodes (LEDs) [24]. These structures of graphene oxide and graphite also have an antibacterial effect on the tubular membrane [25]. We should be aware that these graphene and tubular structures originated from bladder catheters.

In conclusion, 3D observation of the urine bag by LVSEM demonstrated that bacterial or fungal sulfatase and phosphatase attached to the surface of the urine bag oxidize indoxyl sulfate to indigo and indirubin, producing amorphous purple crystals on the surface of the bag.
